# Challenges in Discovering Drugs That Target the Protein–Protein Interactions of Disordered Proteins

**DOI:** 10.3390/ijms23031550

**Published:** 2022-01-28

**Authors:** Judit Oláh, Tibor Szénási, Attila Lehotzky, Victor Norris, Judit Ovádi

**Affiliations:** 1 Institute of Enzymology, Research Centre for Natural Sciences, ELKH, 1117 Budapest, Hungary; olah.judit@ttk.hu (J.O.); szenasi.tibor@ttk.hu (T.S.); lehotzky.attila@ttk.hu (A.L.); 2 Laboratory of Microbiology Signals and Microenvironment, University of Rouen, 76821 Mont Saint Aignan, France; victor.norris@univ-rouen.fr

**Keywords:** protein–protein interaction, drug target, disordered proteins, neurodegeneration

## Abstract

Protein–protein interactions (PPIs) outnumber proteins and are crucial to many fundamental processes; in consequence, PPIs are associated with several pathological conditions including neurodegeneration and modulating them by drugs constitutes a potentially major class of therapy. Classically, however, the discovery of small molecules for use as drugs entails targeting individual proteins rather than targeting PPIs. This is largely because discovering small molecules to modulate PPIs has been seen as extremely challenging. Here, we review the difficulties and limitations of strategies to discover drugs that target PPIs directly or indirectly, taking as examples the disordered proteins involved in neurodegenerative diseases.

## 1. Protein–Protein Interactions (PPIs) as Drug Targets

The classical ‘small molecule’ approach to drug discovery mainly focuses on interactions between small ligands and proteins such as enzymes, ion channels, and receptors, because these proteins typically contain a well-defined ligand-binding site with which small molecules can interact [1,2]. This approach largely ignores protein–protein interactions (PPIs), the reason being that the modulation of PPIs by small molecules is challenging [2,3,4,5]. Instead, PPIs are characterized using methods such as co-immunoprecipitation, pull-down assays, cross-linking, label transfer, far-Western blot analysis, nuclear magnetic resonance, X-ray crystallography and large-scale proteomics [6]. Such characterization is important because PPIs are crucial to protein functions and play pivotal roles in life processes. Not surprisingly, they cause many diseases since they can be responsible for complexes of proteins coming together—or falling apart—when they are not supposed to. Indeed, aberrant PPIs are associated with various diseases, including cancer, infectious and neurodegenerative diseases. However, PPIs are more difficult to therapeutically target than the interactions of globular proteins with other molecules in the cell, because, in general, the interfaces between interacting proteins are (i) highly hydrophobic and larger than the usual receptor-ligand contact areas [7], (ii) flat with few of the grooves that are used in the design of inhibitory molecules, and (iii) have amino acid residues that bind to one another with a high-affinity, which is difficult for small molecules to inhibit [8]. That said, targeting PPIs is increasingly being seen as a promising strategy for the development of new drugs to treat diseases [9,10]. Recently, PPI modulators have entered clinical studies, with some being approved for marketing, indicating that the modulators targeting PPIs have good prospects ([2] and references therein).

## 2. Characteristics of Molecules Targeting PPIs

PPI modulators such as small molecules, peptides, oligonucleotides and antibodies are considered as potential drug candidates [2]. Methods have been developed to screen and identify such modulators that are more effective than those of classical biophysics and medicinal chemistry. These methods, which include allosteric approaches, covalent modifications and fragment-based drug design (FBDD), often require collaborations between different specialists in order to discover, design and optimize chemicals for transmission into cells and modulation of disease-relevant targets. FBDD in particular appears to be a fruitful approach for small molecule drug leads [11]. Fragment libraries—as opposed to typical small molecule libraries—are proving especially useful against targets that are often difficult for small molecules to bind, such as flatter and larger targets without good pockets. Often the binding of a fragment reveals a new chemical space that can be explored in order to obtain a drug lead. 

Compared with small molecule inhibitors, peptide inhibitors have greater affinities and specificities, which makes it easier for them to bind to the target proteins. The major problems with the use of peptide inhibitors are related to their poor membrane permeability and intracellular instability [12]. Extensive studies with the capacity to handle high-dimensional data and complex systems have revealed the value of artificial intelligence to drug discovery and design. Deep learning methods are therefore being used to design drugs with precision. Such design includes the development of drug delivery systems specific to the target. State-of-the-art techniques of drug delivery include antibody-drug conjugates and ligand-targeted conjugates [13]. Molecular dynamics also provides a solid framework to predict the interfacial areas involved in PPIs of pharmaceutical interest. There are interesting examples of how structural, dynamic and energetic information can be combined into efficient strategies which, complemented by experiments, can lead to the design of new small molecules. Encouraging data for targeting key PPIs in angiogenic pathways and antigen-antibody recognition have been reported [14]. Moreover, high-throughput and virtual screenings as well as structure-based design have been suggested as powerful approaches. The identification of “hot spots in which a handful of amino acids contribute a disproportionate amount of the binding” in the interface regions responsible for PPIs is a new promising direction for drug design [15,16]. In fact, several studies of the kinetics and the thermodynamic properties of PPIs have contributed immensely to our understanding of the affinity of these complexes whilst more recent studies on hot spots and interface residues have opened up new avenues in the drug discovery process. This approach has been used in the design of hot spot modulators targeting PPIs with the objective of normalizing such interactions [17].

Current approaches to the development of drugs to modulate ‘intractable’ targets such as PPIs include orthosteric and allosteric methods. Orthosteric approaches, such as those based on protein therapeutics and the binding of small molecules at orthosteric sites, have often suffered from poor performance caused by the difficulties in directly targeting PPI interfaces. Progress in structural biology has led to allosteric regulatory sites remote from the PPI surfaces being considered as targets. Small molecule modulators of PPIs can target allosteric sites so as to alter PPIs [18,19]; these modulators bind to allosteric sites outside the contact surface to cause what is termed allosteric inhibition. Allosteric pockets are topologically distal from the PPI orthosteric sites, and their ligands do not need to compete with the PPI partners, which helps improve the physiochemical and pharmacological properties of allosteric PPI modulators. Moreover, these modulators could stabilize or enhance PPIs by triggering the conformation change of the target protein, thereby enhancing the affinity of the target protein for the other protein. Indeed, the conformational changes induced by the binding of these modulators could increase the number of contact sites at the interface of the two proteins in what is known as allosteric stabilization. These factors help explain why allosteric stabilization is regarded as a potential strategy in PPI drug discovery [20,21]. 

## 3. Disordered Proteins in PPIs as Attractive Drug Targets 

The term ‘proteoform’ was coined to “designate all of the different molecular forms in which the protein product of a single gene can be found, including changes due to genetic variations, alternatively spliced mRNA and post-translational modifications (PTMs)” [16]. These proteoforms are considered to be ‘inducible’ [22]. There are also ‘conformational’ proteoforms due to the presence of intrinsically disordered proteins (IDPs) or structurally flexible regions though it is now believed that any protein exists to some extent as a dynamic conformational ensemble containing multiple proteoforms (hence, an individual, inducible proteoform is also a conformational proteoform). This has led to a paradigm shift with the classical paradigm of “one-gene–one-protein structure–one-function” being replaced by the more general paradigm of a “protein structure-function continuum” [22].

Targeting IDPs, which are frequently implicated in serious diseases, is particularly challenging as they do not have a well-defined structure under physiological conditions [23,24]. The large binding interfaces that are hard to target with small molecules are especially common in the case of the hetero-associations of these disordered proteins. Nevertheless, different experimental and computational approaches have been developed to identify molecules that target IDPs mostly for different types of cancer [25]. The strategies developed for targeting IDPs with small molecules are based on either (i) the screening of chemically diverse or target-oriented compound libraries or (ii) the study of the interfaces and design of molecular candidates capable of binding to the interface. These strategies have shown that those small molecules that are effective target the most hydrophobic regions of the IDPs and hamper the interactions between macromolecules (DNA or protein) and IDPs, which remain in disordered states.

**Figure 1:**
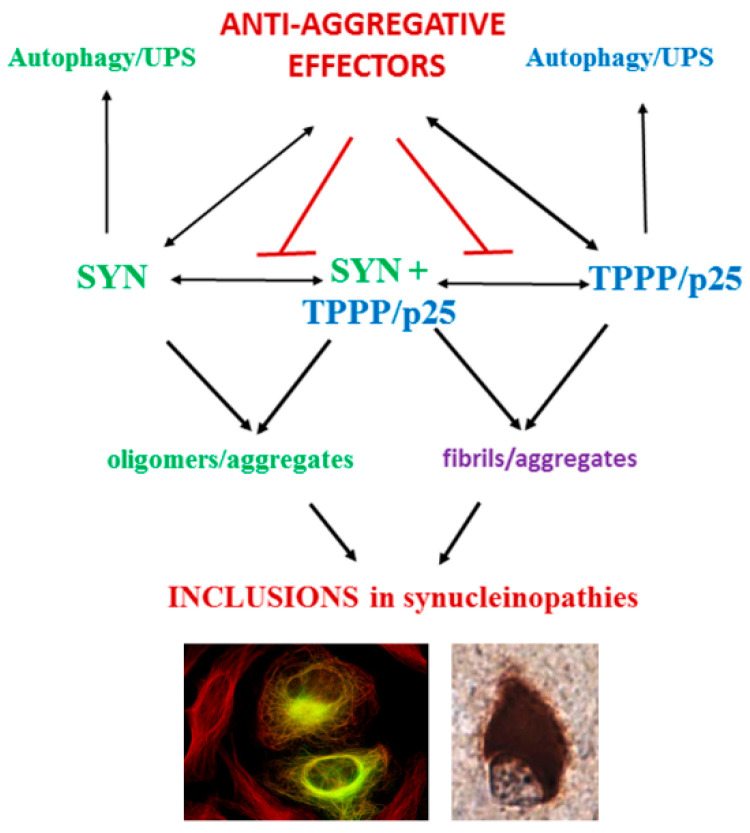
Scheme of the established interactions of SYN and TPPP/p25 leading to inclusion formation in human living cells and brain tissues of patients. UPS: ubiquitin proteasome system. Construction based upon own publications [34,35,36].

IDPs are frequently involved in the pathogenesis of neurodegenerative diseases. They include alpha-synuclein (SYN) and Tubulin Polymerization Promoting Protein (TPPP/p25) in Parkinson’s disease (PD) [26,27], as well as tau and beta-amyloid in Alzheimer’s disease (AD) [28]. Aggregation of these unfolded/misfolded proteins leading to the formation of inclusion bodies as a histopathological hallmark is a common feature in neurodegenerative diseases (Figure 1) [29]. However, the small, soluble oligomeric forms of these proteins are considered the most toxic species [30,31]. Fibrillar seeds of SYN can template the formation of endogenous, toxic oligomers of SYN at an early stage of the disease that can spread throughout the neuron [32]. Cytosolic inclusions such as Lewy bodies have less toxicity and form at the later stage of the disease. They are stable during the course of the disease, and they are eliminated when the neurons that bear them die, consequently, neuronal death is directly related to Lewy bodies [33].

These disordered proteins often display high conformational plasticity (*chameleon proteins*) and multifunctionality (*moonlighting proteins*) and are therefore challenging drug targets [37,38,39]. Although symptomatic treatments are available, there is still a pressing and unmet need for disease-modifying therapies in PD and AD [40,41,42]. Both innovative and well-established strategies for drugs targeting interactions/assemblies of proteins are described below with examples that focus mainly on PD. 

## 4. Strategies for Treating Neurodegenerative Disorders

### 4.1. Direct PPI Targeting

#### 4.1.1. Small Molecules, Peptides

**Figure 2:**
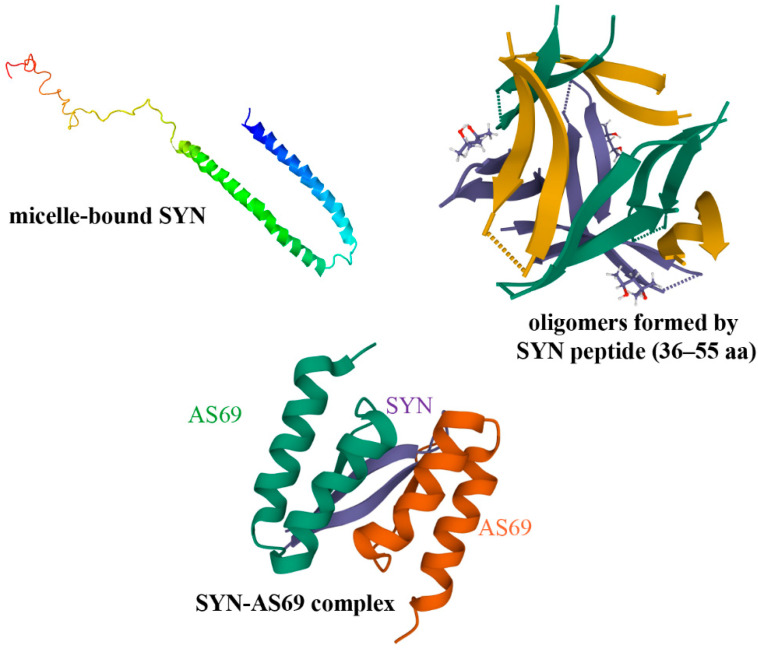
SYN structures: monomeric SYN (Protein Data Bank PDB 1XQ8 [46]), oligomeric SYN (PDB 5F1W [47]) and SYN (purple) complexed with small molecule (AS69 peptide, green and orange) (PDB 4BXL [48]), which inhibits SYN aggregation.

Competition for binding the interface of PPIs by small molecules/peptides can block or modulate the toxic assembly of SYN (Figure 2) [29,43,44,45]. 

Much effort has been focused on stabilizing SYN monomers or inhibiting oligomer formation in order to prevent aggregation [49,50,51]. For example, apomorphine, a nonselective dopamine receptor agonist utilized in PD therapy, inhibited SYN fibrillation; however, it resulted in large, toxic oligomeric species [52].

In addition to the well-characterized pathological oligomerization of SYN, its assembly is also promoted by another brain-specific protein, TPPP/p25 [39]. The occurrence of SYN and TPPP/p25 together are hallmarks of synucleinopathies; these ‘partner proteins’ are co-enriched and co-localized in inclusion bodies in both neurons and oligodendrocytes (OLGs) [27] (Figure 1). These pathological signs are noteworthy since in the healthy brain, SYN and TPPP/p25 occur in neurons and OLGs, respectively [39]. The accumulation and propagation of misfolded proteins aggregates, which can propagate from neuron to neuron in the CNS in a mechanism defined as prion or prion-like, is characteristic of PD, multiple system atrophy and other neuronal disorders ([53,54] and references therein). Moreover, the behavior of SYN has other similarities to that of prions, such as aggregation and conformational changes [55]. Indeed, in the case of synucleinopathies, both the intra- and extracellular transmission of SYN forms between neurons—as well as between neurons and OLGs—are proposed to occur [56,57].

The formation of small, soluble SYN assemblies promoted by TPPP/p25 is likely an early step in pathological processes. Consequently, the targeting of the interface of the pathological SYN-TPPP/p25 complex has been suggested as a new therapeutic strategy [58]. In order to destroy this PPI, the contact surfaces involved in the formation of the SYN-TPPP/p25 complex were identified, and the interface was altered or indeed partially blocked by peptide fragments of the partner proteins [59]. This promising approach may lead to the development of *peptidomimetic foldamers* suitable for pharmaceutical intervention [39].

#### 4.1.2. Allosteric Regulation

**Figure 3:**
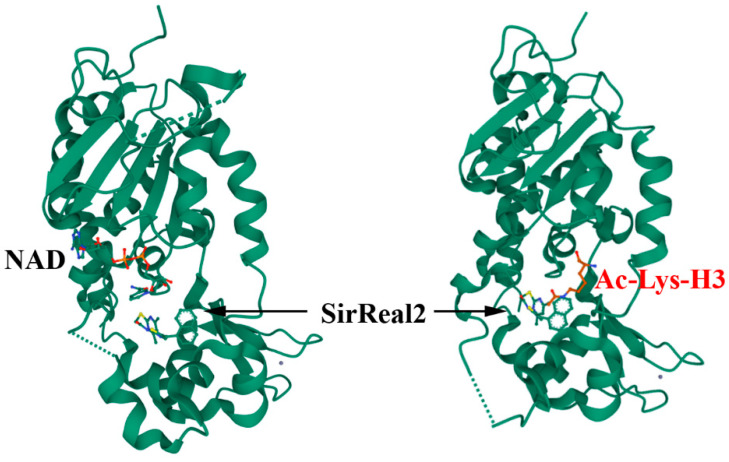
Allosteric regulation: SirReal2 selectively inhibits Sirt2 and functions as a molecular wedge to lock Sirt2 in an open conformation (PDB 4RMG and 4RMH) [62].

The induced-fit theory described by Koshland as allosteric control in 1958 states that the binding of a substrate or other molecule to an enzyme causes a change in the shape of the enzyme so as to enhance or inhibit its activity [60]. Recent technologies have provided a structural basis for these functional consequences. Sirtuin2 (Sirt2) is a tubulin deacetylase implicated in the pathogenesis of cancer and neurodegeneration, and the modulation of its activity is an established strategy for pharmaceutical intervention [61]. Previously, we have shown that a Sirtuin ligand, SirReal2, is particularly effective in inducing a structural rearrangement of the active site that exposes an adjacent binding pocket and that results in hyperacetylation of the microtubule network (Figure 3) [62].

**Figure 4:**
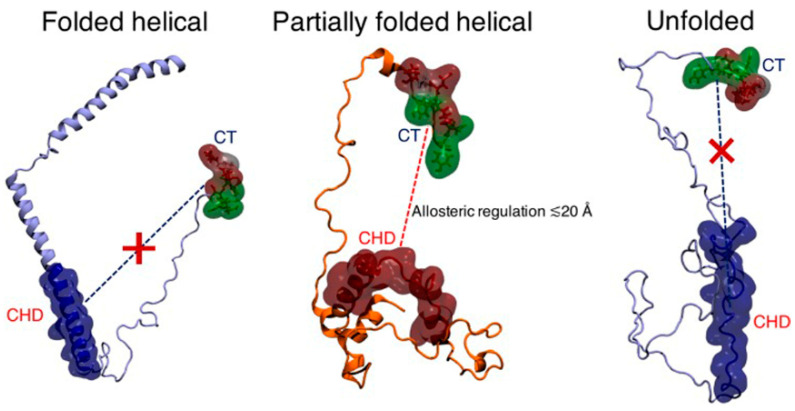
Proposed mechanism of long-range regulation between the tip of the charged C-terminus (CT) and the central hydrophobic domain (CHD) of SYN (from [63]).

Allosteric regulation has been reported for PPIs involving SYN, which is based on targeting SYN outside the interface [63]. In fact, the inter-domain coupling suggests a form of intra-molecular allosteric regulation of the aggregation trigger in the partially folded helical monomers (Figure 4). The aggregation-competence of the SYN is regulated via the dynamics of long-range intra-peptide signaling between two distal sub-domains encompassing the charged C-terminus and the central hydrophobic domain specific for the aggregation-prone partially folded helical state of SYN, but it is absent for the non-aggregating helically folded and the unfolded states; the C-terminus of SYN may therefore be considered as an important target. This finding is consistent with the results of modifying the SYN-TPPP/p25 interaction with a SYN fragment corresponding to the last 14 aa of SYN (the 126-140 SYN peptide) [64].

#### 4.1.3. Oligonucleotide Aptamers

Another way to develop drugs that are highly specific for their targets is to identify those oligonucleotide aptamers that counteract the homologous and heterologous pathological assemblies [65]. Such aptamers, which are regarded as chemical antibodies, are attractive therapeutic agents and are without significant side effects. They have been used successfully in the case of SYN aggregation where two oligonucleotide aptamers obtained using classic magnetic-bead-based aptamer selection were shown to have a capacity to destroy the SYN oligomer in vitro and to be delivered into mouse neurons [66,67]. In addition, our preliminary in vitro experiments have shown that one of these aptamers displays binding affinity to TPPP/p25 and partially inhibits the SYN-TPPP/p25 association. 

#### 4.1.4. PROteolysis TArgeting Chimera (PROTAC) Technology

**Figure 5:**
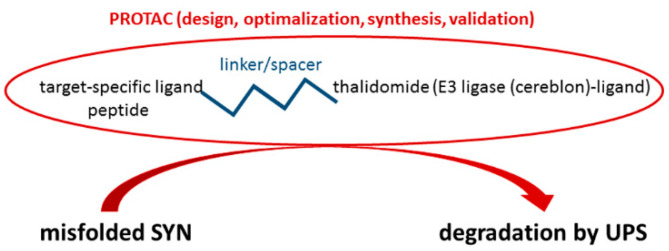
The PROteolysis TArgeting Chimera (PROTAC) technology.

The PROteolysis TArgeting Chimera (PROTAC) technology is proving a way to target therapeutically attractive PPIs by disrupting the undruggable interacting surfaces hence allowing the proteolytic degradation of a partner protein. In this context, important progress has been made by adapting the UPS so as to target selected substrates and prevent PPIs [68]. A chimeric compound of E3 ubiquitin ligase and a SYN-binding motif may allow PD to be treated (Figure 5) [69]. 

This approach has been used to eliminate over-expressed SYN [70,71]. In one case, a peptide degrader was constructed consisting of a cell membrane-penetrating peptide motif, a SYN-binding motif and a chaperone-mediated autophagy-targeting motif [70]. In another case, different SYN-targeting PROTACs were selected (mostly benzothiazole derivatives) that produced significant degradation [71]. 

### 4.2. Indirect PPI Targeting

#### 4.2.1. Regulation at Gene Level: Transcription Factors

The destruction of PPIs via intervention at the gene level is also a promising approach. Disruption of the interactions of transcription factors with the transcription complex results in the downregulation of the selected gene. For example, SRY (sex determining region Y)-box 2, SOX2, is a transcription factor that plays an essential role in early embryonic development and neural differentiation [72]. SOX2 is co-expressed with SYN in the Lewy bodies in the brain of PD patients [73], where SYN is also co-localized with TPPP/p25 [27]. In addition, the increase in SYN is coupled with the decrease in the numbers of SOX2 positive cells in the intracellular inclusions. 

Transcription factor Yin Yang 1 (YY1) is involved in the embryogenesis, differentiation, replication, and proliferation of the CNS [74]. The name reflects its dual effects on transcriptional regulation, since it can either stimulate or repress gene expression. Zinc fingers in the C-terminus of YY1 are responsible for sequence-specific binding to the consensus DNA recognition sequence. A histidine-rich region in the N-terminus serves as a transcriptional activation domain whilst the glycine and lysine-rich middle region forms a transcriptional repression domain. YY1 binds to a single nucleotide polymorphism in the 3’-flanking region of SYN where it stimulates expression of an antisense, noncoding RNA that may be part of a mechanism regulating SYN expression [75]. Recently, the targeting of TPPP/p25 by YY1 has been shown [76], highlighting the involvement of various transcription factors in the pathogenesis of PD. In this neurological disorder, transcription factors that promote inflammation are constitutively upregulated, whilst transcription factors that play a significant role in neuroprotective pathways in brain are substantially downregulated. 

It is clear that a better understanding of the above transcription factors could be used to help the development of targeted drug therapy for neurological disorders. For example, in a mouse model of PD, nanoparticles loaded with miR-124 (a neuronal fate determinant, which has the transcription factor SOX9 as one of its targets) ameliorated motor symptoms [77]. In a rat model, miRNA30-hSNCA (a combination of a microRNA and a short hairpin RNA) was used to silence expression of SYN; however, serious side effects were observed [41,65,78]. Recently, in a transgenic mouse model of PD exosome-mediated delivery of an antisense oligonucleotide (exo-ASO4) has been found to significantly decrease the expression of SYN, to attenuate its aggregation and ameliorate the degeneration of dopaminergic neurons [79]. Targeting the transcriptional factor EB as a master regulator of the autophagy-lysosomal pathway may provide a useful therapeutic tool in neurodegenerative diseases as well [80].

#### 4.2.2. Post-Translational Modifications (PTMs)

IDPs undergo many PTMs that are important in their functioning. SYN, for example, can undergo phosphorylation, nitration, SUMOylation, O-GlcNAcylation, ubiquitination, modification by dopamine and truncation, which can influence its toxicity and aggregation [81]. For example, the phosphorylation of Ser129 of SYN is more frequent in PD than in normal healthy brain, therefore, it can be considered as an unwanted factor, consequently, the prevention of this modification could serve as drug target [82]. The PTMs of the IDPs and their partner proteins accompany the successive conformational changes of the assemblies and help ensure these changes are irreversible. Therapies based on interfering with PTMs include targeting the transmembrane receptor tyrosine kinase, RET, the constitutively active Ser/Thr protein kinase CK2 and the tyrosine phospho-transferase Fyn in order to indirectly affect SYN [83,84,85,86]. 

#### 4.2.3. Other Interactions

There is evidence that calcium-permeable pores formed by small oligomers of amyloid proteins are the primary pathologic species in AD and PD. This pore formation involves two membrane lipids, ganglioside and cholesterol, that physically interact with amyloid proteins through specific structural motifs. Such pores can allow a catastrophic and irreversible entry of calcium ions [87]. This has led to the construction of a small peptide inhibitor that recognizes the gangliosides that are implicated in the initial binding step of amyloid proteins to the lipid rafts of the cell membranes, thereby blocking the neurodegeneration-provoking calcium influx [88,89]. 

Given the propensity of IDPs to undergo PTM, it is likely that IDPs also undergo the covalent addition of poly-(R)-3-hydroxybutyrate (PHB), which modifies a wide variety of proteins [90]. Such modification might be implicated in the IDP translocating to the membrane and even in its calcium channel activity since PHB can assemble with polyphosphate into membranes to form calcium channels [91]. It is likely that some of the residues modified by addition of PHB can also be modified by addition of phosphate [92]; this would mean that compounds promoting or inhibiting kinase and phosphatase activities may also affect the addition or removal of PHB with potentially major consequences for neurodegeneration.

There are also efforts to eliminate SYN aggregates by stimulating macroautophagy, for example using rapamycin, but a lack of specificity and side-effects have prevented using such drugs for PD where long-term treatment would be needed [93]. Encouragingly, however, we have shown recently that that the disruption of SYN assemblies permits the proteolytic degradation of the released SYN (Figure 1) [94].

### 4.3. Multi-Targeting of PPIs

Instead of designing highly selective compounds acting on individual drug targets, the concept of multi-target and combinatorial drug therapies has emerged as an important possibility for the treatment of such complex diseases as PD and AD [95,96]. The combination of drugs with different mechanisms of action may help to increase efficacy and safety. Sometimes, even an individual molecule displays multiple pharmacodynamic actions. For example, amantadine, originally approved as a drug against Asian influenza, combines dopaminergic and glutamatergic properties and is effective in the symptomatic treatment of PD [97]. Protein kinases, such as GSK-3, Fyn, and DYRK1A, are central to neurodegenerative diseases because of their regulatory roles in different signal transduction cascades; multi-target inhibitors of such kinases prevented neurotoxin-induced cell death in in vitro PD models (for references see [98]). Analysis of disease-related genes and PPI networks may help in the development of potential drugs, as shown for AD [99].

**Figure 6:**
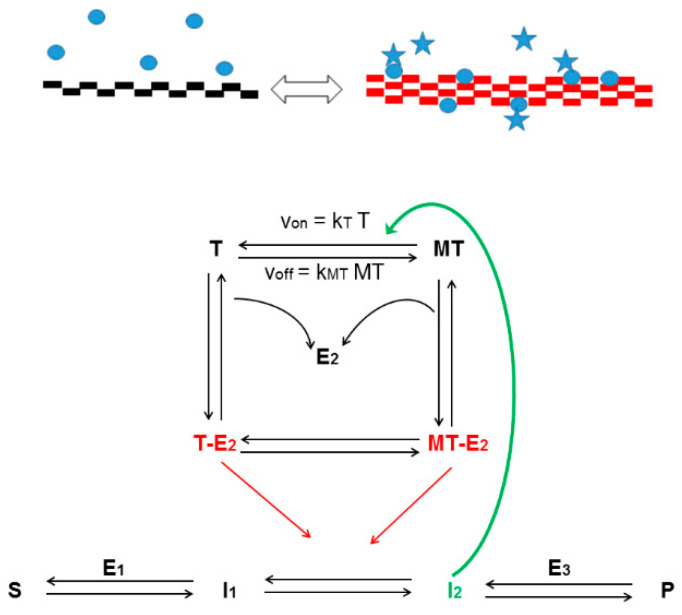
Sensor potency of the enzyme-decorated microtubule network that acts on the dynamics of the microtubule (MT) system (rectangles) and the regulation of the metabolic pathway catalyzed by enzymes (E, blue circles), when these enzymes are either active (presence of star) or inactive. S: substrate, I_1_ and I_2_: intermediates, P: product.

Previously, we have proposed that the highly dynamic cytoskeletal network interacts with enzymes and other multi-functional proteins involved in metabolic or signaling pathways to sense the metabolic state of the cell [100]; the cytoskeletal network therefore comprises multiple targets (Figure 6).

This hypothesis can be extended such that the assembly of IDPs and other macromolecules includes the cytoskeleton. Indeed, many cytoskeleton-binding proteins are IDPs or have *intrinsically disordered regions* and affect cytoskeletal dynamics [101]. For example, the microtubule cytoskeleton, which is essential for cell morphology, cell division, signal transmission, and cellular transport [102], can be considered a multi-target entity. The regulatory proteins with which it interacts include the following IDPs: the microtubule-associated tau, TPPP/p25, and even SYN. The pathological SYN-TPPP/p25 interaction is coupled with the loss of the physiological function of TPPP/p25 [36] such as the modulation of the dynamics and stability of the microtubule network leading to neurological disorders [103]. Therefore, the prevention/destruction of the pathological associations of these proteins can result in the maintenance of physiological interactions.

Understanding and influencing the regulatory complexity of cytoskeletal PPIs are outstanding problems; the biggest obstacle is the lack of high-resolution structures of the interacting proteins. Nevertheless, a few molecules that inhibit or promote PPIs have been synthesized through the rational design method (in addition to those that are of natural origin) [104]. For example, Zampanolide, a 20-membered macrolide from a marine sponge, stabilizes microtubules, arrests cells in mitosis, and inhibits cell proliferation in the low nanomolar range [105,106]. Structural analysis shows that Zampanolide induces changes in the microtubules resulting in an ordered spiral structure, which maintains both the intra- and inter-associations between the microtubule fibrils as well as with the regulatory proteins [107]. 

## 5. Concluding Remarks

The number of PPIs reported in human cells is in the hundreds of thousands and therefore greatly exceeds the number of single proteins, which are the classical targets for pharmacological intervention [108]. PPIs create the dynamic, multiprotein complexes that control metabolic and signaling processes in living organisms in physiological and pathological conditions. Hence, specific and effective modulation of these PPIs in pathological processes is extremely important. Indeed, it should be stressed that modulation of the assembly/disassembly of these complexes through targeting the interfaces of PPIs can offer greater selectivity than the inhibition of the functions of the partner proteins in the PPIs. The development of PPI-based therapies is especially difficult when IDPs are involved in protein associations/assemblies [4] and the strategies reviewed briefly above could open up novel ways of drug discovery leading to the effective treatment of human diseases. In particular, the strategies suggested for PD and other synucleinopathies are likely to prove precious in advancing our understanding of the pathomechanism of these diseases and in generating potential targets for new therapies.

## Data Availability

Not applicable.
